# A Customized Metal Oxide Semiconductor-Based Gas Sensor Array for Onion Quality Evaluation: System Development and Characterization

**DOI:** 10.3390/s150101252

**Published:** 2015-01-12

**Authors:** Tharun Konduru, Glen C. Rains, Changying Li

**Affiliations:** 1 College of Engineering, University of Georgia, 200 D.W. Brooks Dr., Athens, GA 30602, USA; E-Mail: kondurutharun@gmail.com; 2 Department of Entomology, University of Georgia, 2360 Rainwater Road, Tifton, GA 31793, USA; E-Mail: grains@uga.edu

**Keywords:** automation, diseases, gas sensors, onion, post-harvest, volatiles

## Abstract

A gas sensor array, consisting of seven Metal Oxide Semiconductor (MOS) sensors that are sensitive to a wide range of organic volatile compounds was developed to detect rotten onions during storage. These MOS sensors were enclosed in a specially designed Teflon chamber equipped with a gas delivery system to pump volatiles from the onion samples into the chamber. The electronic circuit mainly comprised a microcontroller, non-volatile memory chip, and trickle-charge real time clock chip, serial communication chip, and parallel LCD panel. User preferences are communicated with the on-board microcontroller through a graphical user interface developed using LabVIEW. The developed gas sensor array was characterized and the discrimination potential was tested by exposing it to three different concentrations of acetone (ketone), acetonitrile (nitrile), ethyl acetate (ester), and ethanol (alcohol). The gas sensor array could differentiate the four chemicals of same concentrations and different concentrations within the chemical with significant difference. Experiment results also showed that the system was able to discriminate two concentrations (196 and 1964 ppm) of methlypropyl sulfide and two concentrations (145 and 1452 ppm) of 2-nonanone, two key volatile compounds emitted by rotten onions. As a proof of concept, the gas sensor array was able to achieve 89% correct classification of sour skin infected onions. The customized low-cost gas sensor array could be a useful tool to detect onion postharvest diseases in storage.

## 1. Introduction

Onion is an important vegetable crop around the world [1]. Once harvested, onions are kept in cold storage with a dry and well-ventilated atmosphere for a period ranging from a few weeks to six months before processing. During storage, they are highly prone to a number of post-harvest diseases, most due to infection and mechanical damage during transportation and harvest. It has been a general tradition to hand grade and cull the diseased onions before storing them. These methods often fail to completely remove the infected onions from the lot due to a lack of visual symptoms when onions are still in the early stages of disease development. As a result, certain onions with latent diseases could end up in storage and serve as the vehicle for disease propagation. Differences in the type and concentration of volatiles released by healthy and infected onions were studied by Li *et al.* [2,3]. There is a need in the onion industry for a sensitive and inexpensive gas sensing device that can differentiate between healthy and diseased onions.

Compared to the analytical chemical methods such as GC/MS, the electronic nose (E-nose) technology offers an alternative approach for volatile compounds detection in a rapid and non-destructive way [4,5]. The idea of the E-nose was first proposed by researchers from Warwick University in early 1980s [6]. The proof of concept was presented to use three metal oxide (MOS) gas sensors to identify odors. Since then, there has been a large body of literature in developing and applying the E-nose technology in various fields, such as food and beverage [7,8], environmental monitoring [9,10], and disease diagnosis [11,12]. Major types of gas sensors include MOS, conducting polymer, surface or bulk acoustic wave (SAW or BAW) sensors, and metal oxide field effect transistors (MOSFET). The metal oxide semiconductor (MOS) gas sensors invented by Taguchi in 1960s are one of the earliest commercially available gas sensors with more than 70 types and are perhaps the most widely used gas sensors in the field of E-nose [13]. The MOS sensors are operated at high temperature (50 °C–400 °C) and therefore are insensitive to humidity. Commercial E-noses using MOS sensors include FOX 2000–4000 (Alpha MOS, Toulouse, France), PEN 3 (Airsense Analytics, Schwerin, Germany), and MOSE II (GSG Mess-und Analysengeräte, Bruchsal, Germany). The conducting polymer gas sensor is another widely used and commercially available gas sensor. Similar to the MOS gas sensor, the conducting polymer gas sensor also identifies odors by detecting sensor resistance change, although its operating mechanisms are more complex [13]. One main advantage of using conducting polymer sensors is that they can be operated in ambient room temperature. However, they have relatively slow response, sensitive to water vapor, and tend to drift over time. Commercial E-noses using conducting polymer sensors include Cyranose 320 (Sensigent, Baldwin Park, CA, USA), and Aromascan A32S (Aromascan, Osmetech Inc., Wobum, MA, USA). A surface or bulk acoustic wave (SAW or BAW) sensor detects volatiles compounds by sensing the mass change based on a piezoelectric effect. The zNose 7100 (Electronic Sensor Technology, Newbury Park, CA, USA) is based one SAW sensor. Although there are new technologies such as optical gas sensor [14], they are complementary approaches that enhance the performance of the E-nose [5].

The E-Nose has been extensively studied in food quality and shelf life prediction [15,16]. For instance, the E-nose technology has been proven to be effective in detecting spoilage in various foods such as bakery products, wheat, beef, poultry meat, and milk [5]. In one study, commercial E-nose was used to predict the pleasantness of 22 essential oil odorant mixtures based on the response obtained from the MOSES II E-nose by comparing it with the inputs provided by human subjects [17]. Fish spoilage and freshness evaluation is one area that E-nose technology could be effective and promising because spoiled fish emit unique volatile compounds due to metabolic activities of microorganisms [18,19]. Commercial E-nose technology was also used to evaluate the quality of fruits and vegetables, such as detecting and classifying post-harvest diseases in blueberry fruits [20], quality of apples and oranges [21], and to differentiate various types of *Allium* based on their headspace volatiles and pungency [22]. However, many commercial E-noses are expensive and in some cases unaffordable. The cost of these devices is roughly in the range of $7,000–$80,000. It would be cost prohibitive if multiple E-noses are needed to be deployed in certain settings, such as large onion storage rooms.

The development of a customized E-nose requires a combination of volatile sampling, volatile sensing, electronic control, data recording, and data analysis. There have been several documented efforts to develop a customized E-nose in the laboratory. For instance, a group used MOS (metal oxide semiconductor) sensors and a data acquisition card (USB 6008) to record sensor responses to classify the change in Thai herbs of northern Thailand [23]. E-nose prototypes were fabricated to classify beverages [24] and to perform spoilage classification of beef [25]. A MOS-based gas sensor array was customized for discriminating coffee aromas, essential oils and volatile compounds with different functional groups [26]. Also, based on the concentration of air contaminants such as NO_2_ and CO, indoor air quality was monitored with a customized E-nose [27]. However, these prototypes either do not have on-board computation capabilities or do not have sample delivery system (such as pump and valve). In addition, they are not specific to onion post-harvest disease detection. To fill this gap, a customized gas sensor array was developed, which consists of multiple MOS gas sensors, a gas delivery mechanical system, an automated electronic circuit board, and user friendly software. Due to its low cost, multiple such devices could be deployed in a large onion storage room. The device and the methodology employed in this project could potentially be modified to match the requirements needed for detecting post-harvest diseases in other specialty crops.

The overall goal of this study was to develop a customized gas sensor array for onion postharvest disease detection. Two specific objectives were to: (1) develop the gas sensor array consisting of a mechanical system, electronic system, and software program; (2) characterize and test the developed gas sensor array.

## 2. Overview of the Sensing System

The gas sensor array system was designed to specifically detect volatile profiles of the odor released by onions. The system consisted of mechanical, electronic and software program components. The mechanical component had a gas delivery system to transport the volatiles from the headspace of the sample to the MOS sensors mounted inside a chamber. The electronic component included a circuit board with microcontroller (MCU), memory chip, time keeping chip, and other peripheral devices. Software programs were developed to control the MCU and to interface with the computer. At the MCU level, a program was developed using PIC BASIC PRO (PBP, MicroEngineering Labs, Colorado Springs, CO, USA) to calculate 3 response features for each MOS sensor, namely area under the curve, slope, and relative response. At the computer level, a graphical user interface (GUI) was developed using LabVIEW v8.2 (National Instruments, Austin, TX, USA) to configure the sensor, download and process the data. The construction, characterization and testing of the device are explained in the following sections.

## 3. Mechanical Design

The mechanical components of the device were designed to facilitate delivery of the odor or fresh air to the chamber containing MOS sensors and also to effectively remove the chemical odor from previous experiments. Other considerations included accommodating additional MOS sensors for future studies, low cost and convenient assembly/disassembly.

**Figure 1.**
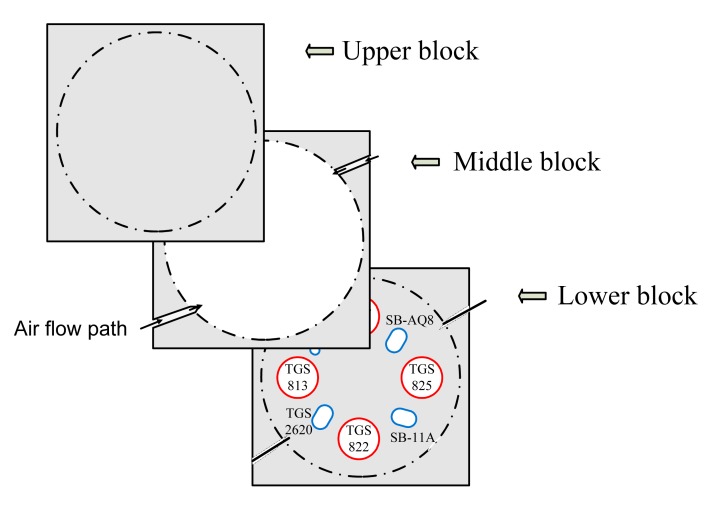
Schematic diagram of the arrangement of three Teflon blocks used for making the gas sensor chamber.

Polytetrafluoroethylene material (PTFE or trademarked name “Teflon”) was used to construct the body of the chamber. Teflon material had several advantages pertaining to the application of this device. It was inert to practically all chemicals and withstands temperature variations. The use of Teflon material also minimized the possibility of sample odor contamination. A square-shaped Teflon block was cut into three 132 × 132 × 19.8 mm sections placed on top of each other after customizing the middle and lower block. Holes (sockets) were bored into the lower block to mount the MOS sensors. Each socket varied in size and shape based on the size of the individual MOS sensor. In the middle block, a circular hole of diameter 112 mm was cut through the block to provide a headspace for the sensors. The arrangement of the three blocks is shown in Figure 1. Any change in MOS sensor size or shape could be easily addressed by replacing the lower block with a suitable design.

The MOS sensors were connected to their respective sockets and plugged into the lower block with their sensing element facing towards the headspace between the upper and lower block, thereby, exposing the sensors to the odor. A simple “leak test” was performed using an air pressure regulator (Norgren Inc., Littleton, CO, USA) and air flow meter (ADM 2000, Agilent technologies, Santa Clara, CA, USA). A minor leak was identified between the blocks and the problem was addressed by placing a rubber fiber gasket wrapped with Teflon tape between the blocks.

The pump and the valve were selected for their oil-free performance, ensuring uncontaminated sample odor or clean airflow to the chamber. Additionally, the pump was selected for its capacity to deliver the sample odor or clean air through the sealed chamber, compact size, pneumatic performance, high durability and pump speed which could be controlled using the MCU. The valve was selected for its type of actuation (*i.e.*, 3-way valve), fluid type (air), compatible port size and flow capacity with the pump. Both the pump and valve components were operated at 12 V DC.

**Figure 2.**
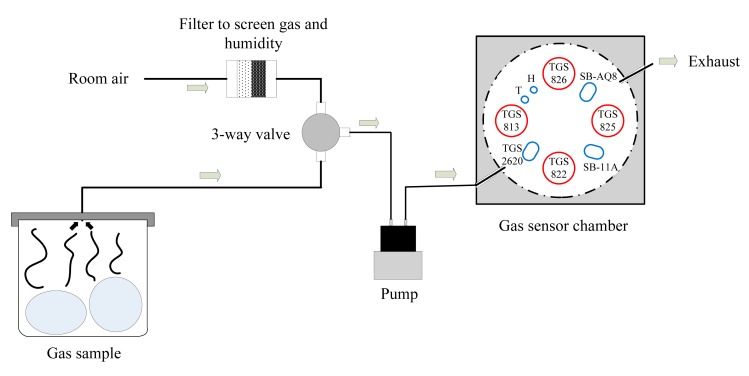
Schematic diagram of the mechanical design of the customized gas sensor array and two air flow paths.

**Figure 3.**
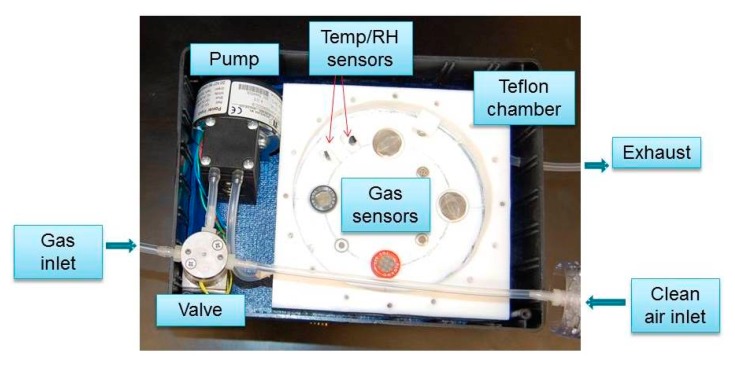
Top view of the mechanical component of the sensing device with top Teflon section removed.

Gas samples were drawn inside the chamber using a pump (NMP 830 KNDC B BLDC motor, KNF Neuberger, INC, Trenton, NJ, USA), and a 3-way valve (225T031, NResearch Incorporated, West Caldwell, NJ, USA) to select one of two pneumatic circuits. One was provided with a filter containing charcoal and desiccant (removes humidity and unwanted odor in the air) to deliver fresh air to clean the chamber. The other air flow path was used to deliver the gas samples of interest to the sensor chamber. Teflon tubing (Outer diameter = 4.76 mm) was used to deliver the gas to the chamber. The schematic diagram and the picture of the mechanical design are provided in Figures 2 and 3, respectively.

A typical sampling process consists of three sequences: Baseline purging, sampling, and purging. “Baseline purging” involved injecting clean filtered air into the chamber preparing the device for the sampling phase. “Sampling phase” involved injecting sample odor into the chamber and the purging phase involved removing the sample odor from the chamber by purging with clean filtered air. This step always followed sampling phase.

## 4. Electronic Design

The electronic circuit performs the sensing of the volatiles, data acquisition (analog to digital conversion), onboard computation (feature extraction from the sensor responses), and data storage. The electronic components and their functions are explained in detail below.

### 4.1. Metal Oxide Semiconductor (MOS) Sensors

The selection of MOS sensors was primarily based on chemical specificity and sensitivity. Secondary parameters included size, cost and power consumption. MOS sensors were not commercially available to detect the specific volatiles released by onions when they are healthy or diseased. Hence, multiple MOS sensors sensitive to a broad range of volatile organic compounds (VOCs) were selected. Volatiles released by infected onions primarily contained compounds belonging to sulfur and aliphatic chemical groups [3]. Aliphatic groups contain compounds such as methane, ethane, propane and butane. MOS sensors sensitive to sulfur, aliphatic compounds and other related compounds were chosen. Table 1 lists the MOS sensors selected and used in the gas sensor array.

### 4.2. Microcontroller

The microcontroller is the essential component in the electronic system. An on-board MCU (PIC 18F4550, Microchip Technology Inc., Chandler, AZ, USA) was selected to perform data acquisition, computations, and control of the various mechanical components in the system. The 40-pin MCU has 35 I/O pins and 13 A/D (Analog/Digital) conversion channels which satisfy the needs of this application. The analog signals (in voltage) from the seven MOS sensors and two temperature and relative humidity sensors were converted to digital signals by the 10-bit A/D converter. Three features were extracted from real-time sensor data to facilitate the following pattern recognition tasks. A more detailed explanation of the feature extraction is provided in the Software section. The MCU controls the operational states of the pump and valve. The 3-way valve was controlled via a metal oxide semiconductor field effect transistor (MOSFET) switch by the MCU because the MCU output (5 V) could not directly drive the valve (12 V). The speed of the pump was operated using a pulse width modulation (PWM) pin available in the MCU. The pump was switched off by providing a “zero” through the PWM pin. Two pump speed selections were programmed into the MCU in which one selection operated the pump at half of its speed and the other at its full speed described as “Low” and “High”, respectively. The data collected from the MOS sensors can be either saved on an onboard memory chip or transmitted to the PC through RS232 communications. The Inter-Integrated Circuit (I2C) and Universal Synchronous/Asynchronous Receiver/Transmitter (USART) communication were essential to perform these tasks. The sampling frequency was 15 Hz with a 20 MHz ceramic oscillator functioning with the MCU.

### 4.3. Memory Chip

The memory chip was selected primarily based on memory space requirements and communication protocol. A 5 V Serial Electrically Erasable PROM (SEEPROM) chip (24AA1025, Microchip Technology Inc.) was used. It had a data storage capacity of 1024 Kbit with a typical writing speed of 3 ms/page using the I2C protocol. With each dataset occupying 24 bytes of space, a total of 5461 datasets could be saved. The data stored in the memory was non-volatile, hence the data was preserved in the chip even if the power supply was cut off. The three features extracted from the sensor responses along with the time stamp were serially written to the memory chip.

### 4.4. Other Peripheral Components

Additional components were needed to perform various tasks. A 3 V DS1302 trickle-charge real time clock chip (Maxim Integrated, San Jose, CA, USA) was used to obtain time information. A RS-232 interface (MAX232IN, Texas Instruments, Dallas, TX, USA) was used to serially communicate with the PC and transfer data from the device to the PC. A parallel LCD was used to display the status of the device.

**Figure 4.**
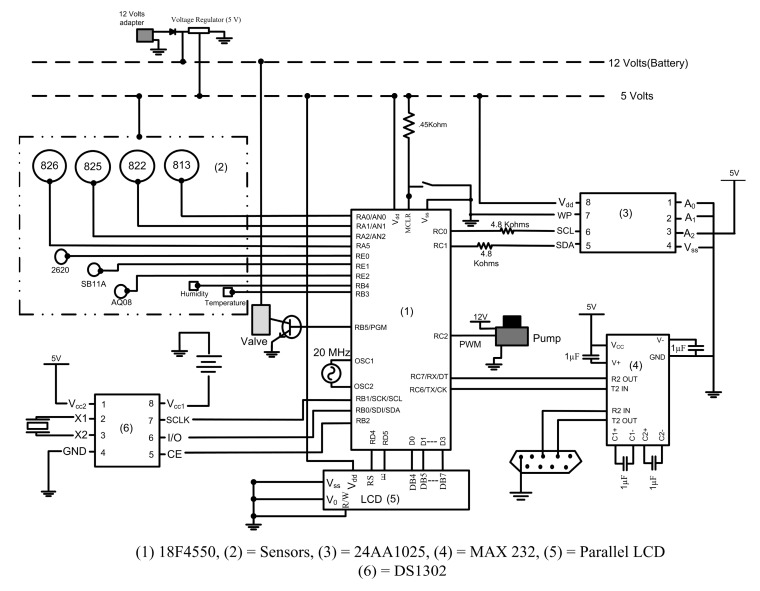
Electronic circuit designed for automation of the customized gas sensor array.

**Figure 5.**
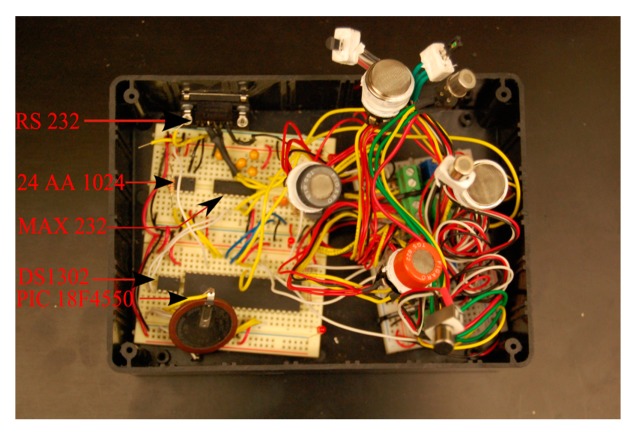
Top view of the electronic circuit designed for gas sensor array.

The entire circuit required three different power supplies. The major portion of the circuit was designed to operate with a power supply of 5 V. The valve and the pump required a voltage supply of 12 V and an external coin battery (3 V) was provided to the DS1302 facilitating the chip to function constantly even when the device was switched off. This eliminated the need for updating the time of the microcontroller every time when the device was switched “on”. The pin connections for the above described components are shown in Figure 4. A top view of the prototype circuit board designed for the gas sensing device is shown in Figure 5.

## 5. Software Design

Two types of software programs were used for the device. First, a MCU program was developed using PIC BASIC PRO (MicroEngineering Labs) to perform the data acquisition, computation, and control of the mechanical devices. Second, user instructions were provided to the MCU using the graphical programming language LabVIEW (v8.2, National Instruments) on the PC. This PC program was used to configure the sensor, download, and process the data.

### 5.1. Microcontroller Program

The MCU program was developed using PIC BASIC PRO (PBP, MicroEngineering Labs) and compiled and downloaded to the MCU via an in-circuit serial programmer (ICSP programmer, MicroEngineering Labs). The MCU could enable the gas sensor array either to function independently without connecting to the computer or with the direct real time control from the computer.

The MCU was programmed to receive instructions from the user to configure its function. The user could provide instructions to collect data, download data, erase data, perform the auto run, forced sampling, and forced purging. When the device was connected to the PC, real-time data was collected in a text (.txt) format. In the event of collecting data for long intervals without human intervention, an “Autorun” function was included that retrieved the default configuration provided by the user. The “Forced sampling” and “Forced purging” selection forced the microcontroller to begin the sampling and purging cycles, respectively. After receiving the user configuration, the MCU was programmed to perform A/D conversions for the seven MOS sensors. The data from the MOS sensors were then used to extract the minimum and maximum values and the time stamps of these values. These parameters were used to calculate the three sensor response features (area under the curve, relative response, and slope). The calculated features along with the time stamp were saved in the memory chip automatically. The absolute time was obtained from a trickle-charge real time clock chip (DS1302). The device was designed and programmed to communicate with the PC through an RS-232 cable connection. This was achieved by setting up the Universal Synchronous/Asynchronous Receiver/Transmitter (USART).

#### 5.1.1. Sensor Configuration

The MCU was operated by the instructions provided by the user via the GUI on the PC. When the device was switched “on” for the first time, the user inputs the time information. The MCU was programmed to calculate the time automatically even when the device was not operating or completely switched “off”. The time information was required from the user only when the coin battery connected to DS1302 was replaced or disconnected.

**Figure 6.**
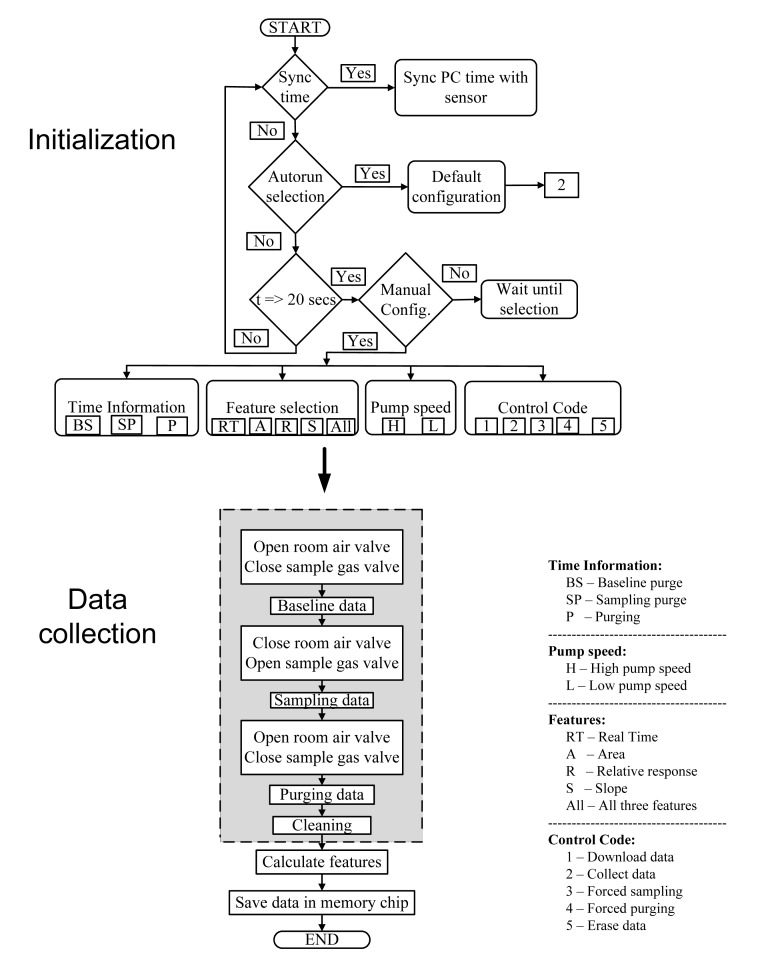
Flowchart of the microcontroller program used for device automation.

The device operated based on multiple user-provided instructions in the form of a string of numerical values. The MCU was coded to respond to each numerical value. The string contained 12 digits: 11 digits were allocated to input command information and the 12th digit for command type. The command enabled the user to select one of four options for feature extraction (1 = area under the curve, 2 = relative response, 3 = slope and 4 = all three features). Pump speed was selected from the two selections: “High speed” and “Low speed”. The duration of the three steps (baseline purge, sampling, and sensor purge) of a sampling procedure can also be determined by the user. These instructions were programmed into the LabVIEW software interface and were provided by clicking the corresponding buttons on the GUI software. The overall structure of the MCU program is illustrated in Figure 6.

#### 5.1.2. Feature Calculation

**Figure 7.**
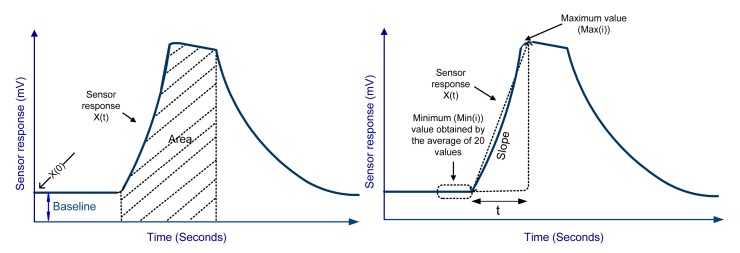
A typical response of a MOS sensor and the features extracted from the response. Similarly, features were calculated from all the MOS sensors.

The device measured the volatile profile of the sample gas under investigation. It was programmed to collect not only the sensors' responses with a 15 Hz sampling frequency, but also maximum value, minimum value, and time taken to reach the maximum value (Figure 7). This information was used to calculate the three different sensor response features; area under the curve, slope and relative response. The feature calculation is discussed below in detail.

Area under the curve was calculated using the trapezoidal rule. An approximate numerical solution (Equation (1)) was used for MCU programming:

(1) $$A\left(i\right)=\sum_{t=0}^{n-1}\frac{1}{2}[f_{i}\left(t\right)+f_{i}\left(t+1\right)]\Delta t$$

where, f_i_(t) = response of sensor “i” at time “t”, f_i_(t + 1) = response of sensor “i” at time “t + 1”, t = current time, *n* = total sampling time (in seconds), A (i) = area under the curve for the MOS sensor “i”, Δ*t*= time interval which was set to 1 s.

Slope feature was computed using the minimum, maximum and the time taken to reach the maximum value. Equation (2) was used to obtain the slope feature:

(2) slope(i)=[max(i)−min(i)]/t

where, slope(i) = slope obtained from the sensor “i”, max(i) = maximum value obtained from the MOS sensor “i”, min(i) = minimum value obtained from the MOS sensor “i”, t = time taken to reach the maximum response in the sampling phase.

The relative response feature was computed using the maximum and minimum values extracted from each MOS sensor's response.

(3) R(i)=[max(i)−min(i)]/min(i)

### 5.2. LabVIEW Program

The graphical user interface (GUI) in the PC was developed using LabVIEW. The purpose of designing this software was mainly to facilitate communication between the user and the device, data processing, downloading and display. The device was configured by the user by making selections on the GUI and transferring them to the MCU in the form of instructions (Figure 7). The instructions were sent to the device through the RS232 cable connected to the device.

**Figure 8.**
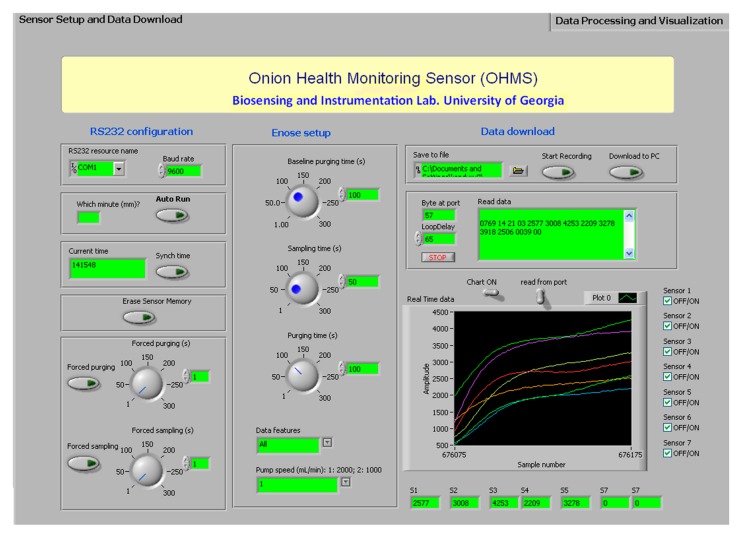
“Sensor setup and data download” tab of the GUI software. Primary functions include: Recording each MOS sensor response, displaying the response, downloading the saved data in the device to the PC, erasing the device memory, synching time, and forced purging/sampling.

The software was designed so the first-time user could easily configure the device. When the device was initiated for the first time, it was programmed to inform the user to sync the time on the PC with the device. This procedure need not be repeated every time the device was switched on due to the independently powered real time clock chip (DS1302). The duration of the three phases (baseline purging, sampling, and purging) can be configured by the user. The type of features and the pump speed were selected from a drop down list. The user started recording by clicking the “Start Recording” button shown in Figure 8.

**Figure 9.**
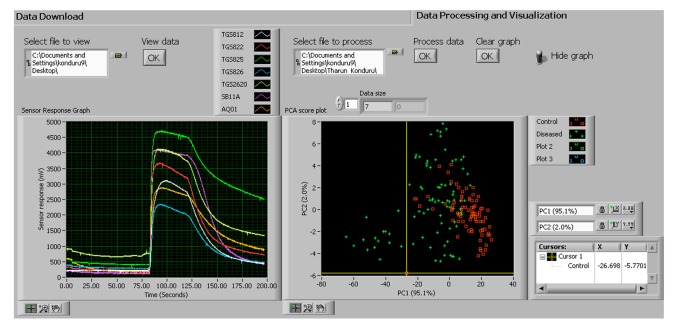
“Data processing and visualization” tab was used to view the collected data in the form of graphs (shown on the left side of the image). Principal component analysis was performed using GUI software. The PCA score plot was obtained along with the principal component values.

The “Download to PC” selection instructs the MCU to transfer the data saved in the memory chip to the PC. A txt format file was created automatically in which the data was saved. Each line contained the serial number, time stamp, area under the curve, slope and relative response values for each MOS sensor. The location of the file could be found in the user specified path of the “save to file” box shown in the “data download” section of Figure 9.

The real time data (in the txt format) can be displayed and analyzed in the “Data processing and visualization tab” (Figure 9). Figure 9 shows the baseline, sampling and the purging phases in the left window. The generated graph was used by the user for quick observation of sensor responses. On the right side of Figure 9, principal component analysis (PCA) was conducted by specifying the path of the file in the “Select file to process” box and clicking “Process data” option. The PCA score plot was used for quick evaluation and diagnosis of the objects being investigated.

## 6. Device Characterization

The mechanical and electronic portion constitutes the physical components of the device. As for the brain of the device, the software was designed and coded with instructions for automation. The following steps involved characterizing the MOS sensors in terms of their response time and sensitivity due to the pump speed and their positioning inside the chamber, as well as their response to volatile compounds of interest.

### 6.1. Determine the Effects of Two Different Pump Speeds on the Sensor Response

It was essential to determine if there was a significant difference in peak response and response speed attained by each MOS sensor for the two different pump speeds (1700 mL/min *vs.* 850 mL/min). A student's t-test was conducted to compare the effect of flow rate for each sensor.

**Figure 10.**
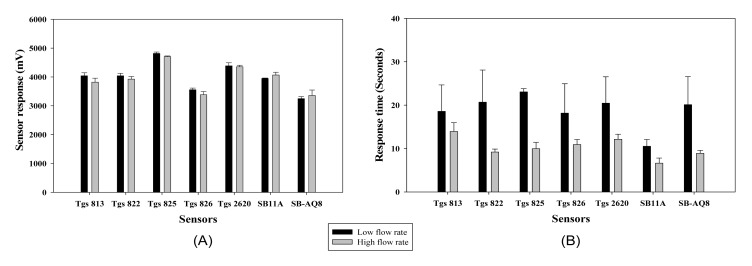
The effect of flow rate on the magnitude of the maximum sensor response (**A**); and the response time (**B**).

Each 0.1 mL of ethanol solution (100%) was injected into three clean 250 mL glass jars (Fisher Scientific, Pittsburgh, PA, USA). The mouths of the jars were covered with clean aluminum foil and sealed by fastening a metal hollow cap on the beaker. The jar was allowed to stand for 30 min to allow complete evaporation of the sample solution into the headspace inside the jar. The sample inlet tubing was placed into the jar approximately 1 to 2 s before the device reached the sampling phase and the sample was drawn into the chamber for analysis. The pump speed affected the rate at which an odor sample was delivered to the MOS sensors resulting in varied response time. The maximum response and the time taken by the MOS sensors to reach maximum response for both pump speeds are presented in Figure 10, respectively.

The MOS sensors did not show a difference in maximum sensor response for the two pump speeds, except for sensor TGS 825. When the time take for each sensor to reach the maximum magnitude is considered, most sensors had faster response time at the higher pump speed (sensors TGS 822, TGS 825, TGS 2620, SB 11A and SB AQ8 showed significant difference at α = 0.1). Based on the results, a default setting of high pump speed was used for all the samples for its fast response and recovery time compared to the lower pump speed.

### 6.2. Determine the Effect of Sensor Response to Sensor Positioning

The MOS sensors were positioned in a circular fashion inside the chamber. Two MOS sensors were positioned near the inlet and outlet holes through which the sample gas enters and leaves the chamber. Due to the potential fluid dynamic effects near the entrance and exit of the air samples, it was important to determine if these MOS sensors observed any entry/exit effects in their responses to the samples. The following test procedure addressed this question.

**Figure 11.**
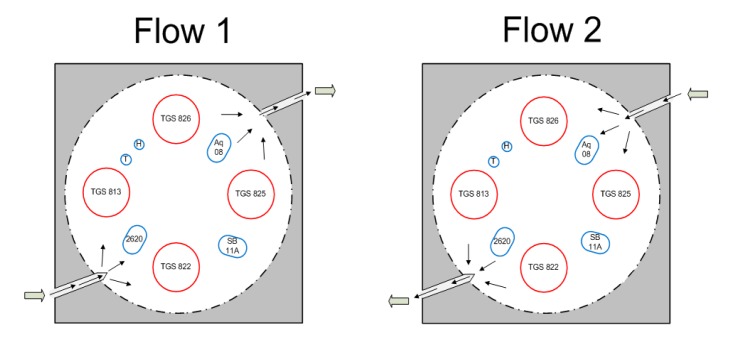
Odor sample flow directions (Flow 1 and Flow 2) into the sensor chamber.

**Figure 12.**
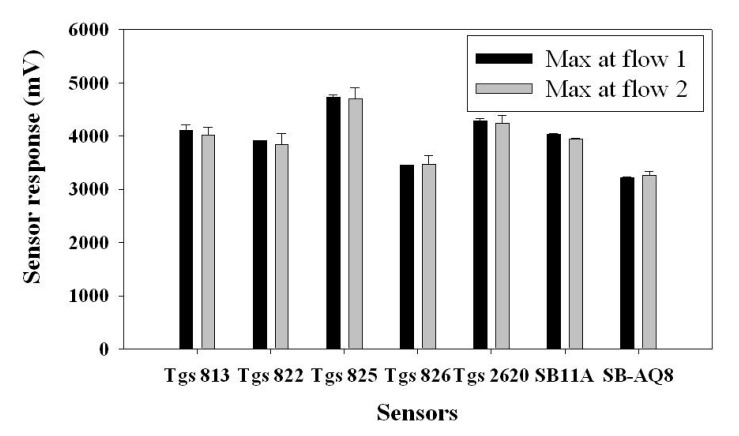
Maximum response (mean ± SD) for 100% ethanolreached by the MOS sensors when tested for two different flow directions.

The same procedure was followed as in previous tests, but the sample was drawn in two different directions represented as “Flow 1” and “Flow 2”. Flow 1 represented the sample flow where the entry point was close to TGS 813 and TGS 825 was the exit point. Flow 2 represented the sample flow where the entry point was TGS 825 and the exit point was close to TGS 813 (Figure 11). The t-test was performed on all the seven sensors in terms of their maximum responses to two flow directions using SAS V9 (SAS, Cary, NC, USA) (Figure 12). Almost all sensors (except SB 11A) showed no significant difference in two flow directions. In other words, a majority of sensors were not sensitive to the position in the gas sensor chamber. In particular, the two sensors located at the entry and exit positions were not affected by the flow direction.

### 6.3. Discrimination of Different Concentrations of Four Chemicals

The operation and sensitivity of the device was tested by exposing it to four chemicals, acetone (ketone, a colorless, flammable organic compound), ethyl acetate (ester, a sweet smelling, colorless organic compound), acetonitrile (nitrile, a simpler form of colorless nitrile), and ethanol (alcohol, a flammable, colorless volatile liquid).

Each of these chemicals with two quantities (20 and 100 μL) was placed in clean 500 mL glass jars (Fisher Scientific). Eight replicates were prepared. The beakers were covered with aluminum foil and sealed by fastening a metal hollow cap before allowing them to stand for 15 min to ensure complete transition of chemical from liquid to gaseous state or until the chemical saturation pressure is reached. The inlet tubing of the sensor was then carefully and immediately placed inside the beaker. For the sensitivity tests, 100 s was set for both baseline purging and sample purging, whereas sampling was done for 35 s. Concentration of the chemical was calculated in parts per million (ppm) as shown in Table 2.

Assuming that the chemical had completely evaporated into the beaker after confirming visually that there is no trace of sample in the jar, the concentration (in ppm) of each chemical was calculated using the following Equation [4]:

(4) $$C=\frac{V_{m}\times V_{l}\times D}{V_{c}\times MW}$$

where, C = concentration of the chemical calculated theoretically in parts per million (ppm), V_m_ = molar volume of an ideal gas at 1 atmosphere of pressure at 25 C (24.45 L/mole) V_l_ = volume of the chemical in liquid form (L) D = density of the chemical (Kg/L) V_c_ = volume of the container (0.5 L) MW = molecular weight of the chemical (g/mole).

**Figure 13.**
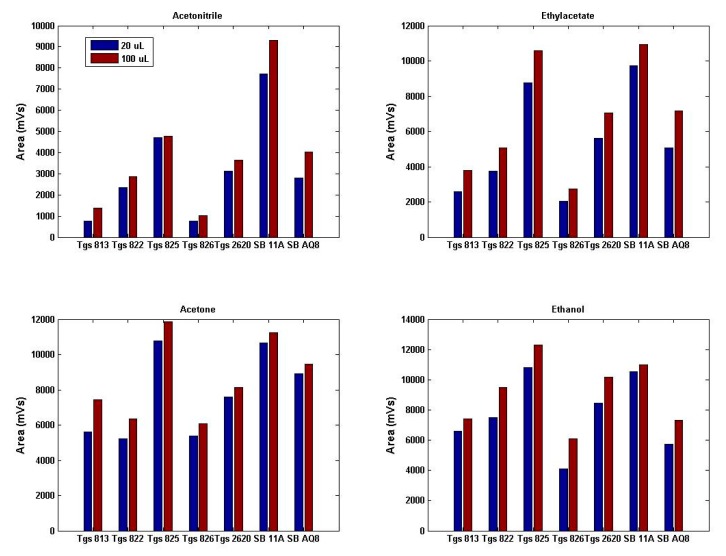
Responses of the gas sensor array to four chemicals at two concentrations.

The smellprints of the four chemicals in two different concentrations showed that the gas sensor array responded differently to four chemicals with two concentration levels (Figure 13). The null hypothesis was tested that there was no significant difference between the same concentrations of all four chemicals and the concentrations within each chemical for all three features (slope, area, and relative response). A MANOVA test was performed using JMP^®^ Pro 9.0.2 (SAS Institute, Cary, NC, USA). Wilk's Lambda statistic was used for testing the significant difference in concentrations within each chemical and between the chemicals. Based on the *p*-value (<0.0001), the null hypothesis was rejected for all comparisons, which indicated that the device responded with significant difference for all three concentrations within the chemical and between the chemicals.

### 6.4. Discrimination of Key Volatiles Released by Infected Onions

Another test was conducted to evaluate the sensor's ability to detect volatile compounds emitted by rotten onions. In the Li *et al.* [3] study, methyl propyl disulfide was released by sour skin and botrytis neck rot diseased onion on the 3rd and 6th day after inoculation whereas 2-nonanone was released by sour skin diseased onion on both the 3rd and 6th day after inoculation. Both chemicals were not released by control healthy onions. The MOS sensors were tested for their response to two different concentrations of these volatiles. 0.5 and 5.0 μL of both chemicals were pipetted into a clean 500 mL beaker. The concentrations of the two chemicals with two volumes are shown in Table 3. Seven and eight replicates were prepared for each concentration of methylpropyl disulfide (90%) (Sigma-Aldrich) and 2-nonanone (>99%) (Sigma-Aldrich) respectively. The beaker was sealed and allowed to stand for 15 min. The collected headspace volatiles were exposed to the gas sensor array for 35 s.

**Figure 14.**
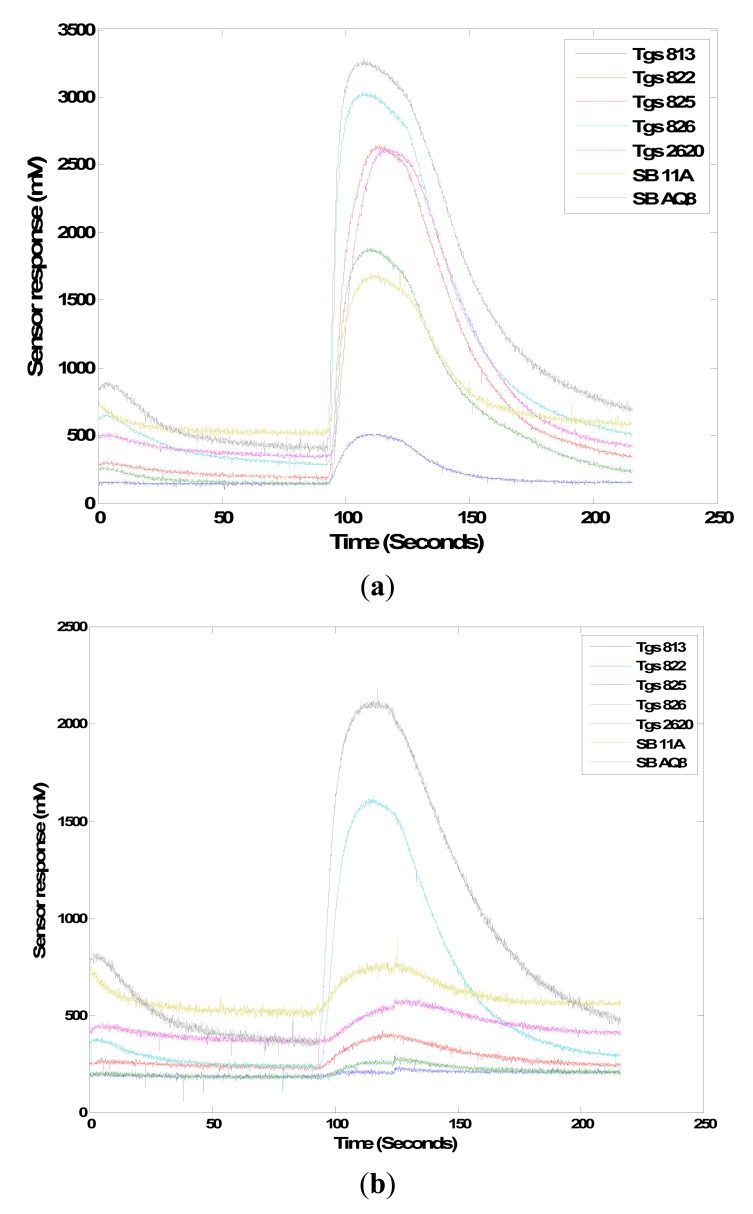
Response obtained from 7 MOS sensors to 0.5 μL of methyl propyl disulfide (**a**); and 2-nonanone (**b**).

It was observed that all seven sensors responded to the two chemicals even at lower concentration level (Figure 14). TGS 826 and SB AQ8 sensors had a higher response to both the chemicals than the other MOS sensors while TGS 813 responded the least. However, single sensors responses may not be indicative of the differentiation power of the sensor array; the collective response (the so called “smellprint”) from the sensor array is more important to examine. The features extracted from the sensor response were statistically analyzed using the MANOVA statistical procedure to determine if the two chemical concentrations were distinguishable. The three features relative response, area and slope obtained for two concentrations of methyl propyl disulfide showed a significant difference with a *p*-value of 0.0012, 0.0003 and 0.0353, respectively. Area and relative response features extracted from two concentrations of 2-nonanone showed significant difference based on *p*-values of 0.0164 and 0.0122 respectively. However, the slope feature extracted from sensor responses to the two concentrations was not significantly different (*p*-value = 0.0678). The results indicate that in majority cases the three features extracted from all the seven sensors' responses could be used to differentiate two concentration levels (10 times difference) of the volatile compounds emitted by sour skin diseases. This could be used to evaluate the severity of the pathogen infection in onions.

### 6.5. Demonstration of Diseased Onion Detection

As a proof of concept, the gas sensor array was tested to classify healthy and sour skin infected onions. Jumbo yellow onion bulbs were purchased from a local grocery store and inoculated by cultures of *Burkholderia cepacia* (causal pathogen for sour skin) using a 3 mL sterile syringe. Sixteen samples with eight control (healthy onions) and eight treatment (sour skin infected onions) were measured using the gas sensor array three times each day from 5 to 6 days after inoculation (DAI). A total of 96 datasets were obtained.

**Figure 15.**
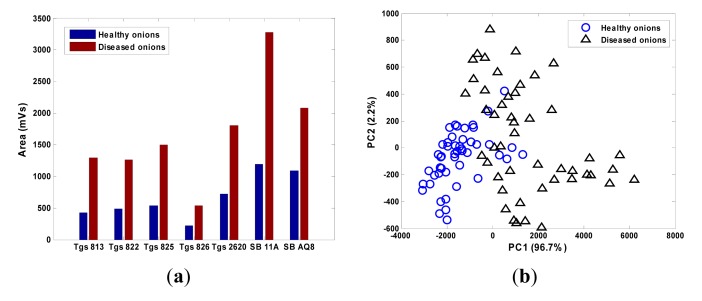
The smellprint (**a**) and PCA score plot (**b**) of healthy and sour skin infected onions.

The raw data from all the MOS sensors were corrected using the differential baseline correction method and the feature was extracted by calculating the area under the section of the sensor response when the device was exposed to the sample odor. The smellprint was obtained by collectively considering the responses from the seven MOS sensors (Figure 15a). The magnitudes of the sensor responses to diseased onions were more than twice of those to healthy onions. The principal component analysis (PCA) score plot showed that the healthy and diseased onions can be largely separated by the first two principal components (Figure 15b).

The linear discriminant analysis (LDA) was performed using Matlab (R2008b, Math Works Inc., Natick, MA, USA) to classify the two groups (control and diseased onion) based on their smellprint. Half of the datasets (*n* = 48) were randomly selected as the training datasets and the other half (*n* = 48) as the validation dataset.

The correct classification rate was 89.6% with two healthy samples misclassified as diseased (4.1% false positive) and three diseased misclassified as healthy (6.3% false negative) (Table 4). The tests proved the efficacy of the customized gas sensor array for diseased onion detection.

Although the MOS sensors are generally thought to be less sensitive to water vapor than other gas sensors such as conducting polymer sensors [13,28], several published papers indicate that water vapor could affect the MOS sensors' conductivity due to the contribution of electrons from the OH group [29–31]. Given this factor, relative humidity should be monitored and considered during the measurement. A relative humidity sensor was included in the gas chamber in our design. Our preliminary data showed that the gas sensors used in this study did not have observable responses to the water vapor. In addition, the humidity is not variable under controlled storage for onions. Therefore, humidity interference should not affect the detection of diseased from healthy onions in controlled storage.

Another well-known limitation of the gas sensors is the sensor drift caused by sensor aging and poisoning. A few studies have shown that machine learning algorithms such as support vector machine and common principal component analysis could be used to correct the sensor drift in the period of 7 months to 3 years, remove faculty sensors, and extend the sensor array's life [32–35]. The sensor drift effect needs to be considered and corrected using similar machine learning methods when the developed gas sensor array is to be deployed in onion storage.

## 7. Conclusions

The custom-built gas sensor array operated well at high pump speeds irrespective of the size of the sample headspace. The location of the MOS sensors within the chamber did not have a significant effect on sensor response. The device was able to differentiate the four chemicals ethanol, acetone, acetonitrile and ethyl acetate when exposed with the same concentrations and was able to differentiate differences in the concentrations within and between the chemicals. The sensor was not only able to detect the presence of the two key volatile compounds emitted by diseased onions, but also to differentiate them at two concentration levels. The gas sensor array was able to achieve 89% classification accuracy when healthy and sour skin infected onions are mixed.

The main contribution of this paper was to develop a low cost customized gas sensor array with an automated gas delivery and data acquisition system to detect volatile compounds emitted by onions. The sensor characterization tests have proven the efficacy of the device for sour skin infected onion detection. The sensor was relatively inexpensive and therefore could be deployed in multiple units in a storage room. It could be modified for other specialty crops for postharvest quality evaluations.

## Figures and Tables

**Table 1.** List of MOS sensors used to build gas sensor array, their gas specificity and manufacturer's reported lower detection limit. Sensors 1–5 were purchased from FIGARO Inc. and sensors 6 and 7 from FIS Inc.

**Sensor No.**	**MOS Sensor**	**Type**	**Specificity**	**Lower Detection Limit (ppm)**
1	TGS 813	Tin dioxide (SnO_2_)	Methane, Ethane, Propane	500
2	TGS 822	Tin dioxide	Organic solvent vapors	50
3	TGS 825	Tin dioxide	Low conc. of H_2_S	5
4	TGS 826	Tin dioxide	Ammonia	30
5	TGS 2620	Tin dioxide	Alcohol and organic solvent vapors	50
6	SB-11A	Tin dioxide	Hydrocarbon	100
7	SB-AQ8	Tungsten trioxide (WO_3_)	VOCs	0.1∼1

**Table 2.** Lists the concentration (ppm) of four chemicals at two different quantities.

	**Ethanol**	**Acetone**	**Ethylacetate**	**Acetonitrile**
20 μL	16,749	13,320	9,957	18,726
100 μL	83,747	66,598	49,785	93,631

**Table 3.** Concentrations (ppm) of two chemicals at two different quantities.

**Volume (μL)**	**Methyl Propyl Disulfide**	**2-Nonanone**
0.5	196	145
5	1964	1452

**Table 4.** Classification confusion table of healthy and sour skin infected onions.

	**Actual Healthy**	**Actual Diseased**
Predicted healthy	21	3
Predicted diseased	2	22
Total	23	25
